# Prevalence of excess body weight and underweight among 26 Chinese ethnic minority children and adolescents in 2014: a cross-sectional observational study

**DOI:** 10.1186/s12889-018-5352-6

**Published:** 2018-04-27

**Authors:** Yanhui Dong, Zhiyong Zou, Zhaogeng Yang, Zhenghe Wang, Yide Yang, Jun Ma, Bin Dong, Yinghua Ma, Luke Arnold

**Affiliations:** 1 Institute of Child and Adolescent Health & School of Public Health, Peking University, No.38 Xueyuan Road, Haidian District, Beijing, 100191 China; 2 South Western Sydney Primary Health Network, Sydney, Australia

**Keywords:** Malnutrition, Excess body weight, Ethnic groups, Children, Adolescents

## Abstract

**Background:**

Little is known regarding the nutritional burden in Chinese ethnic minority children. This study aimed to investigate the epidemiological characteristics of excess body weight and underweight for 26 ethnic groups.

**Methods:**

Data on 80,821 participants aged 7–18 years across 26 minorities, with completed records from a large national cross-sectional survey, were obtained from Chinese National Survey on Students’ Constitution and Health (CNSSCH) in 2014. Excess body weight, underweight and their components were classified according to Chinese national BMI references.

**Results:**

The overall prevalence of excess body weight and underweight among ethnic groups were 12.0% and 14.5%, in which 4.4% and 4.1% of the participants were classified as obese and severe wasting, respectively. Compared with girls, boys showed a higher prevalence of underweight, severe wasting and obesity, but a lower prevalence of excess body weight (*P* < 0.05). Among 26 ethnic groups, Koreans had the highest prevalence of excess body weight (30.4%), while Bouyeis showed the highest prevalence of underweight (25.7%). The ethnic minority groups with high prevalence of excess body weight and underweight were more likely to show high burden of obesity and severe wasting, respectively. However, it is not the case for some groups, such as Miaos and Shuis.

**Conclusions:**

A worrying dual burden of excess body weight and underweight was recognized in Chinese ethnic minority children. Since various characteristics were found among different minorities, the ethnic-specific effort is warranted to improve their nutritional status.

**Electronic supplementary material:**

The online version of this article (10.1186/s12889-018-5352-6) contains supplementary material, which is available to authorized users.

## Background

In the past three decades, child excess body weight (including obesity and overweight) prevalence has risen rapidly among high-, middle- and low-income countries [1–3], including China [4]. A dramatic rise of overweight and obesity had been reported in Chinese children aged 7–18 years old, which had increased over 13-fold in boys and almost 5-fold in girls from 1985 to 2014, which has posed a major public health challenge [5–10]. There is increasing evidence suggesting that childhood obesity and overweight would track into adulthood and account for a substantially elevated future burden of cardiovascular disease [11].

Although much has been written about the epidemic of child obesity, underweight in children and adolescents also generates a considerably major problem, especially in developing countries. According to a WHO report, approximately 16% of children in developing countries are classified as severely malnourished [12]. Malnourished children, particularly those with severe underweight, are not only related with developmental delays and marked cognitive effects [13, 14], but also associated with elevated risk of mortality [15, 16]. However, little is known among Chinese ethnic minority children and adolescents.

China is a multi-ethnic country with 55 ethnic minorities, who make up nearly 8% of the entire Chinese population and have exceeded 100 million by the year 2010 [17]. Though previous studies have reported the obesity epidemic in Chinese children of Han nationality [5, 6, 18–20], it is unclear whether children of ethnic minority groups are experiencing a similar situation.

Using most recent data from the Chinese National Survey on Students’ Constitution and Health (CNSSCH), a national cross-sectional study conducted in 2014, this study investigated the burden of excess body weight, underweight, and their severe situations (obesity and severe wasting, respectively) among children and adolescents aged 7–18 years from 26 ethnic minority groups, and further explored the epidemiological characteristics of the abnormal nutritional status among these ethnicities.

## Methods

### Study population

Data were obtained from the 2014 CNSSCH, which is the largest nationally representative survey of school-aged children designed to investigate their health status in China. The sampling procedures of CNSSCH have been published previously in detail [6]. Briefly, all participants were selected by stratified cluster sampling, that is, sampling took place in classes selected randomly from each grade in the selected schools. The schools in the survey were selected randomly in minority communities from different provinces. The number of each age of children needed to meet the minimum sample size of each ethnic minority in the survey. The population of children and adolescents in other 29 ethnic minority groups was not enough to meet our investigation. Hence, the present study only included boys and girls aged 7–18 years from the following 26 ethnic minorities: Mongol, Hui, Tibetan, Uyghur, Miao, Yi, Zhuang, Bouyei, Korean, Dong, Yao, Bai, Tujia, Hani, Kazak, Dai, Li, Lisu, Va, Shui, Dongxiang, Naxi, Khalkhas, Monguor, Qiang and Salar. They were mainly sampled in the Ethnic Minority Autonomous Regions or Ethnic Minority Autonomous Prefectures. According to the sample size formula of stratified cluster sampling design, the minimum effective sample sizes for excess body weight and underweight in Chinese children of each ethnic minority were 2506 and 1596, respectively. Thus the sample size among ethnic minorities in our study showed appropriate representativeness to investigate disease prevalence of excess body weight and underweight. Participants were involved in this study if they and their parents were of the same ethnic origin and had lived in the respective local area for longer than 1 year. All eligible participants in our study underwent a complete medical examination before data collection and were excluded if they had one or more of the following conditions: (1) serious organ disease (e.g., heart, lung, liver, kidney); (2) abnormal physical development (e.g., pygmyism, gigantism); (3) physical impairment or deformity (e.g., severe scoliosis, rickets, obvious O leg, X leg); or (4) acute disease symptoms (e.g., diarrhea, fever) during the past month and not yet recovered. Of 81,293 participants, 472 participants refused or did not complete the measurements. Thus, our sample size for analysis was 80,821 with the response rate of 99.42%. These surveys were conducted according to the guidelines laid down in the Declaration of Helsinki and has approved by six ministries of China, including the Ministry of Education, General Administration of Sport, Ministry of Health, State Ethnic Affairs Commission, Ministry of Science and Technology, and Ministry of Finance. Informed consent was obtained from both parents and students before they participated in the study and the investigation was carried out following the rules of the Declaration of Helsinki. The project was approved by the Medical Research Ethics Committee of Peking University Health Science Center (IRB00001052–13082).

### Measures

All participants underwent a complete physical examination. Height (cm) and weight (kg) were measured following a standardized procedure by professionals who had passed the training course. The standardized measurement procedure of height and weight in 2014 CNSSCH referred to the anthropometry methods in 2006 WHO Child Growth Standards [21]. Height was measured to the nearest 0.1 cm with portable stadiometers and weight was measured to the nearest 0.1 kg with a standardized scale. All the participants were required to wear only light clothing and stand erect, barefoot and at ease while being measured. Each school-aged child or adolescent independently measured and recorded a complete set of measurements of weight and height, after which the two compared their readings. If any pair of readings exceeded the maximum allowable difference for a given variable (e.g. weight, 100 g; height, 7 mm), both observers once again independently measured and recorded a second and, if necessary, a third set of readings for the height and weight. Both the stadiometers and scales were calibrated before use and similar instruments were used in measurement at all survey sites. Rigid quality control measures were enforced in this survey. All technicians were required to pass the standard measurement test after a rigorous 1-week training course, and all measurements were conducted by the same team of technicians in each province. This would contribute to the minimizing of technical error of measurements. At the end of daily measurements, 3% of the subjects were asked to be measured again. Subjects whose measurements had disparities exceeding the limiting scores were considered as invalid cases. If the proportion of invalid cases was higher than the admissible value, then all the measures of that day were considered as invalid and were measured again.

### Study variables

BMI was calculated as body weight (kg) divided by height (m) squared (kg/m^2^). Since different populations have different growth pattern and fat accumulation [22], excess body weight represented the opposite extreme on the spectrum of adiposity, and was defined as body mass index (BMI) of children ≥ the referent age-and sex- specific 85th centile according to the reference developed by Working Group on Obesity in China (WGOC) [23], including overweight and obesity. Overweight was defined as BMI of children ≥ the referent age-and sex- specific 85th centile but less than 95th centile according to the reference. Obesity was defined as BMI of children ≥ the referent age-and sex- specific 95th centile according to the reference and was defined as the severe condition of excess body weight. Underweight and severe wasting represented the opposite extreme on the spectrum of thinness in children, and was defined as BMI of children ≤ the referent age-and sex- specific percentiles in national screening standard for underweight in Chinese school-aged children and adolescents (WS/T456–2014) [24], which has been shown in Additional file 1: Table S1. A child with extreme underweight was classified as severe wasting, which is a part of the underweight classification.

### Statistical analysis

The distributions of excess body weight and underweight across the age, sex and ethnic groups were presented graphically. To assess the differences in nutritional status among different genders, we used logistic regression to estimate the prevalence odds ratio (POR) for different nutritional status in boys versus girls. The prevalence with 95%CI was adopted to analyze the distribution characteristics and burden of excess body weight and underweight among 26 minority groups. The different burden characteristics of obesity and severe wasting in the 26 ethnic minority groups were analyzed by evaluating the proportion for them in excess body weight and underweight, respectively. All analyses were performed using Stata 12.0 software (College Station, SE) and *P* values less than 0.05 (two-sided) were considered to be statistically significant.

## Results

### Characteristics of the study population

As shown in Additional file 1: Table S2, a total of 40,323 boys (49.9%) and 40,498 girls (50.1%) were involved in this study, and the distribution of sex and age were quite similar among different ethnic groups. The mean ages in boys and girls of different ethnic minority groups ranged from 12.4 to 12.6 years and 12.4 to 12.8 years, respectively.

### Overall prevalence of excess body weight and underweight in 2014

As shown in Table 1**,** the overall prevalence of excess body weight among ethnic minority children and adolescents was 12.0%. The prevalence of obesity, the severe condition of excess body weight, was 4.4%. Compared with girls, boys showed a lower risk of excess body weight but a higher risk of obesity. The overall prevalence of underweight among ethnic minority children and adolescents was 14.5% and the prevalence of severe wasting, the severe condition of underweight, was 4.1%. Compared with girls, boys showed higher risks in both underweight and severe wasting.

**Table 1:** The prevalence of excess body weight, obesity, underweight and severe wasting for boys and girls and their POR in different age groups in 2014, (% or POR, 95% CI)

Classification	Age group/year				Total
	7–9	10–12	13–15	16–18	
Both sexes (%,95% CI)					
N (%)	20,139(24.9)	20,175(25.0)	20,431(25.3)	20,076(24.8)	80,821
Excess body weight^a^	11.6(11.1,12.0)	12.4(11.9,12.8)	11.8(11.4,12.2)	12.1(11.7,12.6)	12.0(11.7,12.2)
Obesity	4.5(4.2,4.8)	5.0(4.7,5.3)	4.3(4.0,4.6)	3.8(3.6,4.1)	4.4(4.2,4.5)
Underweight^b^	16.5(16.0,17.0)	14.2(13.7,14.7)	12.2(11.7,12.6)	14.9(14.4,15.4)	14.5(14.2,14.7)
Severe wasting	6.6(6.2,6.9)	3.9(3.6,4.1)	2.7(2.5,2.9)	3.4(3.1,3.6)	4.1(4.0,4.3)
Boys (%,95% CI)					
N (%)	10,079(25.0)	10,062(25.0)	10,183(25.3)	9999(24.8)	40,323
Excess body weight^a^	12.0(11.3,12.6)	12.4(11.7,13.0)	9.3(8.7,9.8)	10.0(9.4,10.6)	10.9(10.6,11.2)
Obesity	5.2(4.8,5.6)	5.7(5.2,6.1)	4.0(3.6,4.4)	3.4(3.0,3.8)	4.6(4.4,4.8)
Underweight^b^	16.8(16.1,17.5)	16.9(16.2,17.7)	15.6(14.9,16.3)	16.5(15.8,17.2)	16.5(16.1,16.8)
Severe wasting	7.1(6.6,7.6)	4.5(4.1,5.0)	3.1(2.7,3.4)	3.1(2.8,3.5)	4.5(4.3,4.7)
Girls (%,95% CI)					
N (%)	10,060(24.8)	10,113(25.0)	10,248(25.3)	10,077(24.9)	40,498
Excess body weight^a^	11.2(10.5,11.7)	12.5(11.8,13.1)	14.4(13.7,15.0)	14.2(13.6,14.9)	13.0(12.7,13.4)
Obesity	3.8(3.4,4.1)	4.3(3.9,4.7)	4.6(4.2,5.0)	4.2(3.9,4.6)	4.2(4.0,4.4)
Underweight^b^	16.2(15.5,16.9)	11.5(10.9,12.2)	8.8(8.3,9.4)	13.4(12.7,14.1)	12.5(12.1,12.8)
Severe wasting	6.0(5.6,6.5)	3.2(2.9,3.6)	2.3(2.0,2.6)	3.6(3.3,4.0)	3.8(3.6,4.0)
POR^*^ (95%CI)					
Excess body weight^a^	1.09(1.00,1.18)	0.99(0.91,1.08)	0.61(0.56,0.66)	0.67(0.61,0.73)	0.81(0.78,0.85)
Obesity	1.40(1.22,1.60)	1.35(1.19,1.53)	0.87(0.76,0.99)	0.79(0.69,0.92)	1.09(1.02,1.16)
Underweight^b^	1.04(0.97,1.12)	1.56(1.44,1.69)	1.92(1.76,2.09)	1.28(1.18,1.38)	1.38(1.33,1.44)
Severe wasting	1.18(1.06,1.32)	1.44(1.24,1.66)	1.33(1.12,1.58)	0.85(0.73,1.00)	1.18(1.10,1.27)

^*^POR (prevalence odds ratios) was calculated for boys versus girls in different BMI groups

^a^excess body weight involves obesity

^b^underweight involves severe wasting

**Fig. 1:**
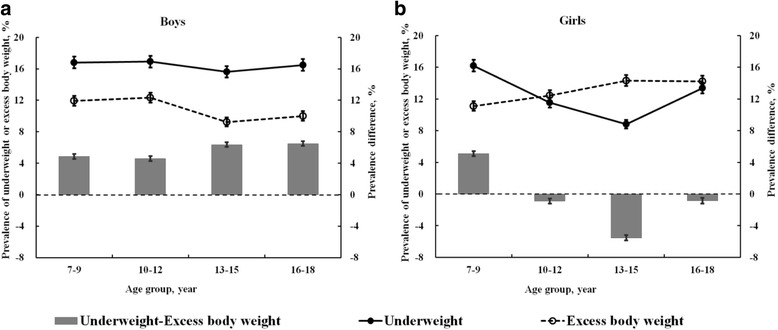
The prevalence of underweight and excess body weight in Chinese boys and girls (**a** and **b**) aged 7–18 years. The solid lines and dash lines represent the prevalence of underweight and excess body weight, respectively. The grey bars represent the differences between underweight and excess body weight. Error bars represent 95% confidence intervals

The prevalence of underweight and excess body weight changed slightly across the age groups in boys, which was higher than that of excess body weight. Among 7–18 years old, a minor increase in the difference between underweight and excess body weight was observed in boys, which changed from 4.6% to 6.5%. A different pattern was detected in girls, whose prevalence of underweight decreased between 7 and 15 years old and increased later, which results in a higher prevalence of excess body weight than that of underweight between 10 and 18 years old (Fig. 1).

### Dual burden of excess body weight and underweight among ethnic minorities

As shown in Table 2, across the 26 minority groups, those with high burden of excess body weight were more likely to demonstrate a low burden of underweight, and vice versa. For instance, Koreans, Kazakhs and Mongolians, whose burden of excess body weight (30.4%, 23.1% and 22.7%, respectively) were high, had the low burden of underweight (5.9%, 4.5% and 4.1%, respectively). In addition, Lis and Shuis, whose burden of excess body weight are relative low (3.4% and 3.6%, respectively), showed a high burden of underweight (25.2% and 25.7%, respectively). However, in some ethnicities, such as Miaos, the burden of both excess body weight and underweight were high (12.8% and 16.0%, respectively). Moreover, a low burden of both excess body weight and underweight was identified in some minorities, such as Kahalkhases and Qiang. These characteristics of dual burden were also presented in Table 2 by sex observing a similar pattern.

**Table 2:** The prevalence for excess body weight and underweight among 26 Chinese ethnic minority children and adolescents

Ethnic minority	Boys		Girls		Total	
	Excess body weight	Underweight	Excess body weight	Underweight	Excess body weight	Underweight
Mongol	19.5(17.9,21.1)	4.8(4.0,5.7)	25.8(24.1,27.5)	3.3(2.7,4.1)	22.7(21.5,23.8)	4.1(3.5,4.7)
Hui	17.5(16.0,19.0)	11.6(10.4,12.9)	14.8(13.4,16.1)	8.8(7.7,9.9)	16.1(15.1,17.1)	10.1(9.3,11.0)
Tibetan	11.8(10.0,13.7)	14.9(13.0,17.0)	12.2(10.4,14.2)	9.2(7.6,11.0)	12.0(10.7,13.4)	12.1(10.8,13.4)
Uyghur	8.8(7.8,9.9)	11.5(10.4,12.7)	13.9(12.7,15.3)	11.1(10.0,12.4)	11.4(10.5,12.2)	11.3(10.5,12.2)
Miao	9.2(7.6,11.0)	19.0(16.9,21.4)	16.4(14.3,18.6)	12.9(11.1,15.0)	12.8(11.5,14.2)	16.0(14.6,17.5)
Yi	7.3(6.0,8.7)	21.2(19.1,23.4)	9.8(8.3,11.4)	11.6(10.0,13.4)	8.5(7.5,9.6)	16.4(15.0,17.8)
Zhuang	14.2(12.9,15.6)	15.1(13.8,16.5)	10.6(9.5,11.9)	12.7(11.4,14.0)	12.4(11.6,13.4)	13.9(13.0,14.8)
Bouyei	5.3(4.1,6.8)	28.1(25.6,30.8)	6.8(5.4,8.3)	22.8(20.5,25.3)	6.1(5.1,7.1)	25.5(23.7,27.3)
Korean	31.3(29.4,33.3)	7.1(6.0,8.2)	29.4(27.5,31.3)	4.7(3.8,5.6)	30.4(29.0,31.7)	5.9(5.2,6.6)
Dong	9.8(8.1,11.6)	18.8(16.7,21.2)	10.7(9.0,12.6)	12.2(10.4,14.2)	10.2(9.0,11.5)	15.5(14.1,17.0)
Yao	8.7(7.1,10.5)	22.0(19.6,24.5)	11.7(9.9,13.8)	20.4(18.1,22.9)	10.2(9.0,11.5)	21.2(19.5,22.9)
Bai	11.6(9.9,13.4)	20.0(17.8,22.2)	10.5(8.9,12.3)	12.8(11.0,14.7)	11.0(9.9,12.3)	16.4(15.0,17.8)
Tujia	17.1(15.0,19.2)	14.0(12.2,16.1)	15.2(13.3,17.3)	11.2(9.5,13.0)	16.1(14.7,17.6)	12.6(11.4,14.0)
Hani	4.1(3.1,5.3)	19.1(17.0,21.3)	7.7(6.3,9.2)	14.0(12.2,16.0)	5.9(5.0,6.8)	16.6(15.2,18.0)
Kazak	19.2(17.1,21.3)	6.0(4.8,7.3)	26.8(24.5,29.2)	3.0(2.2,4.0)	23.1(21.4,24.6)	4.5(3.8,5.3)
Dai	7.0(5.7,8.6)	30.0(27.5,32.5)	7.4(6.1,9.0)	18.8(16.7,21.0)	7.2(6.3,8.3)	24.4(22.7,26.1)
Li	3.5(2.6,4.5)	29.0(26.7,31.4)	3.4(2.6,4.4)	21.5(19.4,23.6)	3.4(2.8,4.1)	25.2(23.7,26.8)
Lisu	3.1(2.2,4.2)	19.5(17.4,21.7)	6.8(5.5,8.3)	12.4(10.7,14.3)	5.0(4.2,5.9)	16.0(14.6,17.4)
Va	6.4(5.2,7.9)	21.9(19.7,24.2)	14.7(12.8,16.7)	14.5(12.6,16.5)	10.6(9.4,11.8)	18.2(16.7,19.7)
Shui	1.5(0.9,2.4)	28.0(25.5,30.7)	5.8(4.5,7.2)	23.4(21.0,25.9)	3.6(2.9,4.5)	25.7(23.9,27.5)
Dongxiang	6.1(4.8,7.5)	20.2(18.1,22.5)	6.9(5.6,8.4)	25.1(22.8,27.5)	6.5(5.6,7.5)	22.7(21.1,24.3)
Naxi	11.2(9.6,13.1)	15.8(13.9,17.9)	13.4(11.6,15.4)	9.8(8.2,11.5)	12.3(11.1,13.6)	12.8(11.5,14.1)
Khalkhas	4.0(3.1,5.2)	12.4(10.7,14.3)	10.2(8.7,11.9)	7.6(6.3,9.1)	7.1(6.2,8.1)	10.0(8.9,11.2)
Monguor	2.1(1.4,3.1)	20.6(18.5,22.9)	5.7(4.5,7.2)	13.4(11.6,15.4)	3.9(3.2,4.7)	17.0(15.6,18.5)
Qiang	7.0(5.7,8.5)	14.7(12.9,16.7)	11.7(10.1,13.5)	8.5(7.0,10.1)	9.4(8.3,10.5)	11.6(10.4,12.8)
Salar	4.9(3.8,6.2)	14.4(12.5,16.4)	5.2(4.1,6.6)	20.9(18.8,23.2)	5.0(4.2,5.9)	17.6(16.2,19.1)

### Burden of obesity and severe wasting across 26 ethnic minority groups

**Fig. 2:**
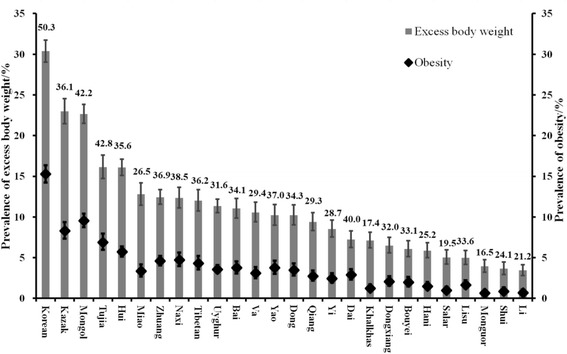
The proportion of obesity in excess body weight among 26 ethnic minority children and adolescents. Grey bars represent the prevalence of excess body weight and black dots represent the prevalence of obesity. Error bars represent their 95% confidence intervals. Numbers represent the proportion of obesity in excess body weight

The burden of excess body weight and underweight was further investigated by analyzing the proportion of their severe conditions, obesity and severe wasting, respectively. As shown in Fig. 2, a majority of the ethnic groups with high prevalence of excess body weight were more likely to show elevated prevalence of obesity. For instance, the Korean group had a high prevalence of excess body weight (30.4%), with about 50.3% of those with excess body weight classified as obese. However, some minorities with low burden of excess body weight, such as Bais (prevalence of excess body weight was 11.0%), also had a high prevalence of obesity (40.0% of Bais with excess body weight were classified as obese).

**Fig. 3:**
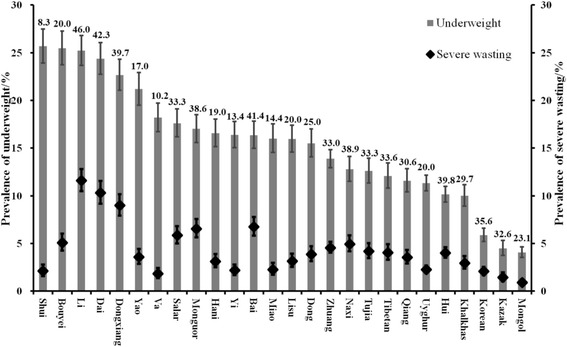
The proportion of severe wasting in underweight among 26 ethnic minority children and adolescents. Black dots represent the prevalence of severe wasting and grey bars represent the prevalence of underweight. Error bars represent their 95% confidence intervals. Numbers represent the proportion of severe wasting in underweight

A similar pattern was also found in underweight and severe wasting (Fig. 3). Although ethnic minority groups with high prevalence of underweight were more likely to show elevated prevalence of severe wasting, some ethnicities with high burden of underweight had low prevalence of severe wasting, such as Shui (prevalence of underweight was 25.7%; 8.3% of those with underweight were classified as severe wasting). In contrast, some groups with relatively low prevalence of underweight had high proportion of those classified as severe wasting, such as Bais (prevalence of underweight was 16.4%, but 41.4% of those underweight were classified as severe wasting).

## Discussion

Among 80,821 children and adolescents across 26 ethnic minority groups, we found that boys had lower burden of excess body weight, but increased burden of obesity, underweight and severe wasting, compared with girls. The results also indicated a higher prevalence of underweight in boys than that of excess body weight, however, a different result was detected in girls aged 10 to 18 years who showed a higher prevalence of excess body weight than malnourished. The present study additionally revealed that the characteristics of the burden of abnormal nutritional status vary among different ethnic minority groups. It is of great significance to analyze ethnic difference in the burden of excess body weight and underweight, which is helpful to develop policies and interventions for improving the nutrition status in these young populations.

The burden of excess body weight in boys was lower than that of girls, though the prevalence of obesity was higher in boys. Some studies conducted in western countries, such as the United States, Canada, France, Germany, and Italy, have revealed the sex difference and illustrated that boys were more likely to be obese than girls [25, 26]. Although the mechanisms are not fully demonstrated, previous studies showed the risk of being obese differs by sex during puberty periods [27, 28]. Accumulation of fat was more likely to appear in girls after the timing of pubertal onset because of hormone secretion. We found the prevalence of excess body weight and obesity in boys and girls were different before and after the onset of puberty. For example, boys showed higher risks of excess body weight and obesity before 12 years, but lower risks of excess body weight and obesity after 12 years than girls (Table 1). The age of 12 years was the approximate time of puberty in boys and girls. Thus, we speculated that puberty could be the reason why different risks for excess body weight were found between boys and girls before and after 12 years. These variations in fat accumulation could also partly explain why girls had lower prevalence of underweight than boys in this study. A similar finding was found in Han children and adolescents during the same period as well [29, 30]. However, the mechanism has still not been fully demonstrated, and further study is warranted to clarify it.

Our results indicated the burden of excess body weight was higher than that of underweight in minority girls. This results were consistent with that in Han girls, which showed the prevalence of excess body weight and underweight were 14.6% and 8.9%, respectively [7]. Compared with Chinese Han children in other studies using the same definitions [31], the prevalence of excess body weight was lower in Chinese ethnic minorities who additionally showed higher prevalence of underweight across the age groups. Though Chinese children are presenting a faster transition from underweight to excess body weight [32], the speed of shift is higher in Chinese Han than that in ethnic minorities. Thus scientific intervention policy aimed to prevent unhealthy nutritional transition in ethnic minorities is needed. Nutrition improvement interventions in minority areas may have contributed to the declined prevalence of underweight [33]. However, a conflicting result was identified in boys whose burden of excess body weight was lower than that of underweight. Previous studies have demonstrated that the demand of nutrients and calories in boys is higher than that in girls during puberty [28, 29]. As a consequence, if the supply is not adequate, boys are more likely to be malnourished. In addition, China is currently experiencing a transition from a history of underweight to a very rapid increase of obesity [34, 35], and a considerable heterogeneity in the timing of the transition from underweight to excess body weight provides variation in weight change between boys and girls [36, 37]. Therefore, the existence of excess body weight and underweight in ethnic minority children may be not be synchronous in both sexes. As the observation of dual burden of underweight and excess body weight, both problems become major health issues affecting Chinese ethnic minority children and warrant more attention to each issue simultaneously.

The present study revealed different ethnic characteristics by analyzing 26 Chinese minority population groups. The Korean, Mongolian and Kazakh groups with the lowest underweight prevalence were more likely to be obese, which may be related with their dietary behavior. Mongolian and Kazakh are traditional nomadic ethnicities, and they make a living by animal husbandry. Koreans live in the northeast region of China, where the climate is characterized by a winter lasting more than 6 months in duration with the temperature below freezing. As a result, Koreans are typically accustomed to eat a lot of meat to maintain body temperature. Surveys in these minority groups also found that the frequency and amount of meat intake in children and adolescents were much higher than their counterparts of other minorities [33]. The Shui, Bouyei, Li, and Dai children ranked in the top four minority groups for underweight, and had the lowest prevalence of excess body weight. High proportions of severe wasting in underweight were also detected in Li and Dai children, which could be attributed to their relative low socio-economic levels. These four minorities live in southwest undeveloped mountain area with a low standard of living. These results indicated the importance of ethnic-specific policies, and different approaches are warranted to improve the nutritional status among various minorities.

This study showed that Chinese ethnic minority children are facing a dual burden of excess body weight and underweight. Our results have public health implications for policy making and targeted interventions among ethnic minorities. On one hand, both excess body weight and underweight in childhood are associated with adulthood chronic diseases, including diabetes and cardiovascular diseases [38–40]. The customized implementation of nutrition improvement program and obesity intervention has a potential to reduce the risk of these chronic disease in minority areas. On the other hand, the ethnic differences in dual burdens among various minorities suggest minority-specific policies are warranted to reduce these burdens effectively. Exploring the influence factors for dual burden in ethnic minority groups could be helpful to find their advantages and disadvantages and further develop a suitable nutrition intervention programs for local minority young population.

This study has a large sample size and involves more than 80,000 ethnic minority children and adolescents. It has strengths for addressing the nutrition problems in these populations. However, several limitations of this study should also be noted. Firstly, there are 55 ethnic minorities in China, but only 26 ethnic minority groups were included in our study. Studies conducted in other minorities are desired to address the health problems among other ethnic minorities. Second, the data of this study were derived from a cross-sectional study, and we did not analyze the risk factors of excess body weight and underweight. However, the epidemiologic characteristics of abnormal nutrition status in this study were developed from the latest available data with large sample size, which could help us to depict the nutrition problems in these minority populations. Third, BMI was used in our results to indicate nutritional status of children, which may do not distinguish between lean and fat mass. Other more precise markers could account for more of the BP trends, like dual energy X-ray absorptiometry and MRI. However, BMI is regarded as the measurement method highly correlated with more precise measures of adiposity and thinness, and it is widely used in epidemiological studies. Other precise markers are not suitable for our surveys with such a large sample size because they are resource intensive measurements.

## Conclusions

Chinese ethnic minority children are facing the dual burden of excess body weight and underweight. Although excess body weight, including obesity, is becoming an emerging health problem in these young people, underweight remains a critical problem. However, the characteristics of the dual burden vary among different minorities. Minority-specific policies are warranted to improve the nutritional status and relieve the worrying dual-burden in Chinese ethnic minority children and adolescents.

## Additional file

Additional file 1: **Table S1.** Showed the national reference of underweight components (severe wasting and mild wasting) for Chinese boys and girls aged 7–18 years. **Table S2.** showed the characteristics of participants among 26 ethnic minority groups in 2014. (DOCX 35 kb)

## Abbreviations

BMI: Body mass index
CNSSCH: Chinese National Survey on Students’ Constitution and Health
WGOC: Working Group on Obesity in China
