# Supplementary material for: Prevalence of excess body weight and underweight among 26 Chinese ethnic minority children and adolescents in 2014: a cross-sectional observational study
Source: BMC Public Health. 2018 Apr 27;18:562. doi: 10.1186/s12889-018-5352-6 (PMC5923026; doi:10.1186/s12889-018-5352-6)
Supplement: Supplementary file 1 — Table S1. Showed the national reference of underweight components (severe wasting and mild wasting) for Chinese boys and girls aged 7–18 years. Table S2. showed the characteristics of participants among 26 ethnic minority groups in 2014. (DOCX 35 kb) [file 12889_2018_5352_MOESM1_ESM.docx]

| **Table S1** The national reference of underweight components (severe wasting and mild wasting) for Chinese boys and girls aged 7-18 years | | | | | |
| --- | --- | --- | --- | --- | --- |
| Age | Boys | |  | Girls | |
|  | Severe wasting | Mild wasting |  | Severe wasting | Mild wasting |
|  |  |  |  |  |  |
| 7.0~ | 13.5 | 13.6~13.9 |  | 13.0 | 13.1~13.4 |
| 7.5~ | 13.5 | 13.6~13.9 |  | 13.0 | 13.1~13.5 |
| 8.0~ | 13.6 | 13.7~14.0 |  | 13.1 | 13.2~13.6 |
| 8.5~ | 13.6 | 13.7~14.0 |  | 13.1 | 13.2~13.7 |
| 9.0~ | 13.7 | 13.8~14.1 |  | 13.2 | 13.3~13.8 |
| 9.5~ | 13.8 | 13.9~14.2 |  | 13.2 | 13.3~13.9 |
| 10.0~ | 13.9 | 14.0~14.4 |  | 13.3 | 13.4~14.0 |
| 10.5~ | 14.0 | 14.1~14.6 |  | 13.4 | 13.5~14.1 |
| 11.0~ | 14.2 | 14.3~14.9 |  | 13.7 | 13.8~14.3 |
| 11.5~ | 14.3 | 14.4~15.1 |  | 13.9 | 14.0~14.5 |
| 12.0~ | 14.4 | 14.5~15.4 |  | 14.1 | 14.2~14.7 |
| 12.5~ | 14.5 | 14.6~15.6 |  | 14.3 | 14.4~14.9 |
| 13.0~ | 14.8 | 14.9~15.9 |  | 14.6 | 14.7~15.3 |
| 13.5~ | 15.0 | 15.1~16.1 |  | 14.9 | 15.0~15.6 |
| 14.0~ | 15.3 | 15.4~16.4 |  | 15.3 | 15.4~16.0 |
| 14.5~ | 15.5 | 15.6~16.7 |  | 15.7 | 15.8~16.3 |
| 15.0~ | 15.8 | 15.9~16.9 |  | 16.0 | 16.1~16.6 |
| 15.5~ | 16.0 | 16.1~17.0 |  | 16.2 | 16.3~16.8 |
| 16.0~ | 16.2 | 16.3~17.3 |  | 16.4 | 16.5~17.0 |
| 16.5~ | 16.4 | 16.5~17.5 |  | 16.5 | 16.6~17.1 |
| 17.0~ | 16.6 | 16.7~17.7 |  | 16.6 | 16.7~17.2 |
| 17.5~18.0 | 16.8 | 16.9~17.9 |  | 16.7 | 16.8~17.3 |
| Note: The cut-off values of severe wasting and mild wasting were calculated based on individual body mass index (BMI). Underweight included severe wasting and mild wasting. | | | | | |

| **Table S2.** The characteristics of participants among 26 ethnic minority groups in 2014 | | | | | | | | | | | |
| --- | --- | --- | --- | --- | --- | --- | --- | --- | --- | --- | --- |
| Ethnic minority | Province (region) | N | Boys, n (%) |  | Age group/year, n (%) | | | |  | age (Mean ± SD) | |
|  |  |  |  |  | 7-9 | 10-12 | 13-15 | 16-18 |  | Boys | Girls |
| Mongol | Inner Mongolia | 5107 | 2542(49.8) | | 1265(24.8) | 1261(24.7) | 1296(25.4) | 1285(25.2) | | 12.5±3.4 | 12.5±3.4 |
| Hui | Ningxia | 5308 | 2570(48.4) | | 1249(23.5) | 1193(22.5) | 1479(27.9) | 1387(26.1) | | 12.5±3.3 | 12.8±3.4 |
| Tibetan | Tibet | 2418 | 1214(50.2) | | 605(25.0) | 608(25.1) | 605(25.0) | 600(24.8) |  | 12.5±3.5 | 12.5±3.4 |
| Uyghur | Xinjiang | 5740 | 2869(50.0) | | 1438(25.1) | 1434(25.0) | 1433(25.0) | 1435(25.0) | | 12.5±3.5 | 12.5±3.5 |
| Miao | Guizhou | 2394 | 1197(50.0) | | 599(25.0) | 595(24.9) | 600(25.1) | 600(25.1) |  | 12.5±3.5 | 12.5±3.5 |
| Yi | Sichuan | 2863 | 1432(50.0) | | 712(24.9) | 714(24.9) | 719(25.1) | 718(25.1) |  | 12.5±3.5 | 12.5±3.4 |
| Zhuang | Guangxi | 5267 | 2628(49.9) | | 1313(24.9) | 1307(24.8) | 1360(25.8) | 1287(24.4) | | 12.5±3.5 | 12.5±3.4 |
| Bouyei | Guizhou | 2395 | 1198(50.0) | | 600(25.1) | 600(25.1) | 596(24.9) | 599(25.0) |  | 12.5±3.5 | 12.5±3.5 |
| Korean | Jilin | 4546 | 2282(50.2) | | 1192(26.2) | 1191(26.2) | 1165(25.6) | 998(22.0) |  | 12.3±3.3 | 12.3±3.3 |
| Dong | Guizhou | 2398 | 1200(50.0) | | 599(25.0) | 600(25.0) | 600(25.0) | 599(25.0) |  | 12.5±3.5 | 12.5±3.5 |
| Yao | Guangxi | 2251 | 1119(49.7) | | 548(24.3) | 564(25.1) | 572(25.4) | 567(25.2) |  | 12.5±3.4 | 12.6±3.4 |
| Bai | Yunnan | 2627 | 1312(49.9) | | 653(24.9) | 657(25.0) | 657(25.0) | 660(25.1) |  | 12.5±3.4 | 12.5±3.5 |
| Tujia | Hunan | 2592 | 1296(50.0) | | 652(25.2) | 640(24.7) | 657(25.4) | 643(24.8) |  | 12.5±3.5 | 12.5±3.4 |
| Hani | Yunnan | 2637 | 1318(50.0) | | 659(25.0) | 660(25.0) | 658(25.0) | 660(25.0) |  | 12.5±3.5 | 12.5±3.5 |
| Kazak | Xinjiang | 2868 | 1436(50.1) | | 716(25.0) | 713(24.9) | 720(25.1) | 719(25.1) |  | 12.5±3.5 | 12.5±3.5 |
| Dai | Yunnan | 2626 | 1308(49.8) | | 659(25.1) | 658(25.1) | 659(25.1) | 650(24.8) |  | 12.5±3.4 | 12.5±3.5 |
| Li | Hainan | 3034 | 1507(49.7) | | 746(24.6) | 772(25.4) | 774(25.5) | 742(24.5) |  | 12.5±3.4 | 12.5±3.4 |
| Lisu | Yunnan | 2638 | 1318(50.0) | | 660(25.0) | 658(24.9) | 659(25.0) | 661(25.1) |  | 12.5±3.5 | 12.5±3.5 |
| Va | Yunnan | 2639 | 1320(50.0) | | 660(25.0) | 664(25.2) | 654(24.8) | 661(25.1) |  | 12.5±3.5 | 12.5±3.5 |
| Shui | Guizhou | 2391 | 1192(49.9) | | 598(25.0) | 594(24.8) | 600(25.1) | 599(25.1) |  | 12.5±3.5 | 12.5±3.5 |
| Dongxiang | Gansu | 2599 | 1300(50.0) | | 625(24.1) | 715(27.5) | 613(23.6) | 646(24.9) |  | 12.6±3.4 | 12.5±3.4 |
| Naxi | Yunnan | 2636 | 1318(50.0) | | 658(25.0) | 658(25.0) | 660(25.0) | 660(25.0) |  | 12.5±3.5 | 12.5±3.4 |
| Khalkhas | Xinjiang | 2825 | 1417(50.2) | | 708(25.1) | 716(25.4) | 687(24.3) | 714(25.3) |  | 12.5±3.5 | 12.5±3.5 |
| Monguor | Qinghai | 2620 | 1315(50.2) | | 652(24.9) | 649(24.8) | 666(25.4) | 653(24.9) |  | 12.5±3.4 | 12.5±3.5 |
| Qiang | Sichuan | 2762 | 1379(49.9) | | 700(25.3) | 684(24.8) | 685(24.8) | 693(25.1) |  | 12.5±3.5 | 12.5±3.5 |
| Salar | Qinghai | 2640 | 1336(50.6) | | 673(25.5) | 670(25.4) | 657(24.9) | 640(24.2) |  | 12.4±3.5 | 12.4±3.4 |
| Total |  | 80821 | 40323(49.9) | | 20139(24.9) | 20175(25.0) | 20431(25.3) | 20076(24.8) | | 12.5±3.4 | 12.5±3.4 |
